# Ceftazidime dosage regimen recommendations in burn patients based on a Monolix population pharmacokinetic study

**DOI:** 10.1186/cc10673

**Published:** 2012-03-20

**Authors:** S Ruiz, JM Conil, B Georges, F Ravat, T Seguin, P Letocart, O Fourcade, S Saivin

**Affiliations:** 1 Hôpital Rangueil CHU Toulouse, France; 2 Centre hospitalier St Joseph et St Luc, Lyon, France; 3 Institut Fédératif de Biologie CHU Toulouse, France

## Introduction

The aim of our present work was to predict in burn patients the best adapted ceftazidime dosage regimen to obtain a serum target of 40 to 100 mg/l taking into account the influence of patients' characteristics on ceftazidime pharmacokinetics (PK).

## Methods

A Monolix population PK model was developed and validated in 70 burn patients with *Pseudomonas aeruginosa *infection. Monte Carlo simulations (*n *= 1,000) were performed to explore the appropriateness of different dosage regimens in burn patients. Target concentrations to achieve were defined as a 40 to 100 mg/l steady-state concentration interval. The recommended dosage was chosen as the minimum dose providing the maximum of patients in this interval.

## Results

A two-compartment model described ceftazidime disposition. Serum creatinine and age were identified as covariates of ceftazidime clearance. Age also influences the volume of distribution. The simulations showed that the common dosage regimens of 6 g/day did not allow achieving the desired target interval. This was achieved with continuous administration dosage regimens varying between 8 and 16 g/day in the youngest patients. Whatever the dosage regimen, the age and the serum creatinine, the mean highest percentage of patients reaching the 40 to 100 mg/l target interval was 76.43 ± 2.13% (range: 65.1 to 80.1%) (Table 1).

**Table 1:** Recommended ceftazidime dosage regimen

	Target = 40 mg/l							
**Creatinine** **(μmol/l)**	**20 years**	**30 years**	**40 years**	**50 years**	**60 years**	**70 years**	**80 years**	**90 years**

30	16 g	16 g	16 g	16 g	14 g	12 g	12 g	10 g
40	16 g	16 g	16 g	14 g	12 g	12 g	10 g	10 g
50	16 g	16 g	16 g	12 g	12 g	10 g	10 g	8 g
60	16 g	16 g	14 g	12 g	12 g	10 g	8 g	8 g
70	16 g	14 g	12 g	12 g	10 g	10 g	8 g	8 g
80	14 g	14 g	12 g	10 g	10 g	8 g	8 g	8 g
90	14 g	14 g	12 g	10 g	10 g	8 g	8 g	6 g
100	14 g	12 g	10 g	10 g	8 g	8 g	8 g	6 g
120	12 g	10 g	10 g	8 g	8 g	6 g	6 g	6 g
140	10 g	10 g	8 g	8 g	6 g	6 g	6 g	4 g
160	10 g	8 g	8 g	6 g	6 g	6 g	4 g	4 g

Recommended ceftazidime dosage regimen after a 2 g loading dose required to reach a steady-state concentration between 40 and 100 mg/l in the highest percentage of typical burn patients in function of serum creatinine and age.

## Conclusion

This study highlights the peculiarities of ceftazidime pharmacokinetics in burn patients with high interindividual variability. Age and serum creatinine significantly influence the ceftazidime disposition. These covariates must be used to propose the first doses of ceftazidime. The required dosage regimens are higher than in other ICU patients and doses between 4 and 16 g/day are proposed.

